# Role of miRNA-145, 148, and 185 and Stem Cells in Prostate Cancer

**DOI:** 10.3390/ijms23031626

**Published:** 2022-01-30

**Authors:** Donatella Coradduzza, Sara Cruciani, Caterina Arru, Giuseppe Garroni, Aleksei Pashchenko, Mosab Jedea, Silvia Zappavigna, Michele Caraglia, Evzen Amler, Ciriaco Carru, Margherita Maioli

**Affiliations:** 1 Department of Biomedical Sciences, University of Sassari, 07100 Sassari, Italy; donatella.coradduzza@libero.it (D.C.); sara.cruciani@outlook.com (S.C.); cate.cate91@gmail.com (C.A.); giugarroni21@gmail.com (G.G.); alexpko@seznam.cz (A.P.); carru@uniss.it (C.C.); 2 Institute of Biophysics, 2nd Faculty of Medicine, Charles University, V Uvalu 84, 150 06 Prague, Czech Republic; amler@seznam.cz; 3 Department of Precision Medicine, University of Campania “L. Vanvitelli”, Via de Crecchio 7, 80138 Naples, Italy; mosab.jedea@unicampania.it (M.J.); silvia.zappavigna@unicampania.it (S.Z.); michele.caraglia@unicampania.it (M.C.); 4 Student Science, s.r.o., Nar. hrdinu 279, 190 12 Prague, Czech Republic; 5 Center for Developmental Biology and Reprogramming (CEDEBIOR), Department of Biomedical Sciences, University of Sassari, Viale San Pietro 43/B, 07100 Sassari, Italy

**Keywords:** miRNAs, stem cells, cell proliferation, PCSCs, prostate cancer

## Abstract

MicroRNAs (miRNAs) are small non-coding RNA molecules that play a role in cancer linked to the regulation of important cellular processes and pathways involving tumorigenesis, cell proliferation, differentiation, and apoptosis. A lot of human miRNA sequences have been identified which are linked to cancer pathogenesis. MicroRNAs, in prostate cancer (PC), play a relevant role as biomarkers, show a specific profile, and have been used as therapeutic targets. Prostate cancer (PC) is the most frequently diagnosed cancer in men. Clinical diagnoses among the gold standards for PC diagnosis and monitoring are prostate-specific antigen (PSA) testing, digital rectal examination, and prostate needle biopsies. PSA screening still has a large grey area of patients, which leads to overdiagnosis. Therefore, new biomarkers are needed to improve existing diagnostic tools. The miRNA expression profiles from tumour versus normal tissues are helpful and exhibit significant differences not only between cancerous and non-cancerous tissues, but also between different cancer types and subtypes. In this review, we focus on the role of miRNAs-145, 148, and 185 and their correlation with stem cells in prostate cancer pathogenesis. MiR-145, by modulating multiple oncogenes, regulates different cellular processes in PC, which are involved in the transition from localised to metastatic disease. MiR-148 is downregulated in high-grade tumours, suggesting that the miR-148-3 family might act as tumour suppressors in PC as a potential biomarker for detecting this disease. MiR-185 regulation is still unclear in being able to regulate tumour processes in PC. Nevertheless, other authors confirm the role of this miRNA as a tumour suppressor, suggesting its potential use as a suitable biomarker in disease prognosis. These three miRNAs are all involved in the regulation of prostate cancer stem cell behaviour (PCSCs). Within this contest, PCSCs are often involved in the onset of chemo-resistance in PC, therefore strategies for targeting this subset of cells are strongly required to control the disease. Hence, the relationship between these two players is interesting and important in prostate cancer pathogenesis and in PCSC stemness regulation, in the attempt to pave the way for novel therapeutic targets in prostate cancer.

## 1. Introduction

MicroRNAs (miRNAs) are small non-coding RNA molecules (<22 nt) that play a role in influencing multiple mRNAs. To date, more than 1400 human miRNA sequences have been identified, many of which are linked to cancer pathogenesis. They modulate protein expression levels in a manner complementary to their target mRNA. MicroRNAs (miRNAs) are small non-coding RNA molecules (<22 nt) that contribute to influencing multiple mRNAs. Their relevance in cancer is linked to the regulation of important cellular processes and pathways involving tumorigenesis, cell proliferation, differentiation, and apoptosis. It has been suggested that microRNAs in prostate cancer (PC) play a relevant role as biomarkers and show a specific profile, and more recently have been used as therapeutic targets in several studies. Correlations with miRNA expression in solid tumours were frequently found in breast, bladder, and pancreatic cancer.

Prostate cancer is the most common cancer in men, whose risk of development increases with age. Given the prevalence of subclinical forms, especially after the age of 50, PSA screening is currently recommended for all men over 50 years, according to the European Society of Medical Oncology (ESMO) recommendations from 2020. Currently, the gold standards for PC diagnosis and monitoring are prostate-specific antigen (PSA) testing, digital rectal examination, and histopathological evaluation of prostate needle biopsies. Screening and regular follow-up are important steps in reducing cancer mortality, in which PSA determination plays an important role. PSA screening can reduce PC mortality, but there is still a large grey area of patients with threshold values of the marker. The downside of screening is that these studies lead to overdiagnosis and the identification of threshold conditions that increase the burden on the health care system and the subsequent excessive implementation of invasive diagnostic and therapeutic interventions [1]. Despite their clinical value, these criteria have limitations in terms of case recognition, prediction of disease outcomes, and the management of clinical decisions. Therefore, new biomarkers are needed to improve existing diagnostic, prognostic, and therapeutic management strategies [2]. In this regard, highly specific molecular markers, such as metabolic factors of certain tumour cells, or molecular markers, such as microRNAs or specific miRNA signatures, would be very useful.

In PC, screening of miRNA profiles from tumour versus normal tissues reported inconsistent patterns, showing occasional upregulation and generally downregulation of miRNAs. Although about 50 miRNAs have been linked to PC, few have been shown to be related to disease onset. Cancer development affects all processes in the cell, including the regulation of miRNA expression. MiRNAs suppress gene expression based on their complementarity to a part of one or more regulatory regions in two main ways: by blocking protein translation or by accelerating mRNA degradation through polyadenylation inhibition [3]. The regulation of gene expression by miRNAs is important for cellular processes, as abnormalities lead to the development of various diseases of the heart, nervous system, inflammatory autoimmune diseases, and cancers, including PC [4].

In cancer research, terms such as oncomiR and tumour suppressor miRNAs have been known for a long time, but due to the importance of the context of miRNA expression (tissue, organ, stage, etc.) unambiguous separation of miRNAs is not possible [5]. The growing body of evidence has shown that miRNA expression profiles differ not only between cancerous and non-cancerous tissues, but also between different cancer types and subtypes [4,6]. Expression of miRNAs also differs between early and late-stage cancers and depends on the aggressiveness of the disease [7]. The comparison of cancerous and non-cancerous prostate tissue led to the identification of a specific miRNA expression profile that changes with disease development. An important discovery was made when miRNAs were detected in body fluids, such as blood [8,9], urine [10], and seminal fluid [11]. Since then, numerous studies describing miRNAs as potential biomarkers, both in biological fluids and tissues from PCa patients, were published. Several miRNAs were found to be dysregulated in PCa, but only some were described in a huge number of independent studies and were reported in Table 1. It is noteworthy that certain miRNAs were shown to be contradictorily deregulated by several scientific groups (Table 1). The absence of reproducibility has been ascribed to differences in sample types, processing, methods, and analysis [12,13]. 

Analysis of published studies to identify specific miRNAs or signatures that could act as biomarkers is often complex and, to reach a reasonable level of confidence, is very challenging. Song et al. in their meta-analysis excluded a third of published studies on miRNAs in PCa because of an absence of key information [12]. In this review, we focused our interest on three miRNA, miRNA-145, miRNA-148, and miRNA-185, that were dysregulated in different multiple studies both in plasma/serum and tissues. More than 60% of the studies reported in Table 1 (based on a meta-analysis by Song et al. and Bertoli et al.) suggest that the expression levels of miRNA-145 are downregulated in PC and together with miR-148, and miR-185 is reported to be associated with various cancers including PCa because of targeting stem cell pluripotency, differentiation, and autophagy [14,15]. In a previous study (under review), we observed the behaviour of these three miRNAs in the plasma of patients with PC precancerous lesions and prostatic hypertrophy. In this research, we demonstrated that the different expressions of miR-145, miR-148, and miR-185 allowed the discrimination between BPH, PL, and PC patients. MiRNAs could be a useful help to overcome inherent limitations of the prostate-specific antigen (PSA) biomarker in clinical practice. In recent research, a key role of prostate cancer stem cells (PCSCs) in PC has been highlighted. Prostate stem cells (PSCs) are the target cells for oncogenic transformation and may play a role in the initiation of PC as well in the development of resistance to anti-cancer treatments. In this light, the specific targeting of this cancer subpopulation has become pivotal for the further development of more active and successful strategies in the treatment of this disease. In this review, we discuss the current evidence supporting an important role of specifically identified miRNAs in this subject and its possible correlation with PCSC stem cells in PC initiation and development. 

### 1.1. MiR-145

MiR-145 is known to be a tumour suppressor and its expression has been found to be reduced in most human malignancies. MiR-145 is a major downregulated miRNA in PC. MiR-145 has been shown to be downregulated in localised PC and is involved in the transition from localised to metastatic disease [1,2,16,17]. It has also been experimentally demonstrated to target the insulin receptor substrate-1 (IRS-1) [18]. 

In 2016, a meta-analysis by Pashaei et al. collected a large amount of data showing that deregulation of many miRNAs causes increased proliferation, migration, invasion, and apoptosis [19]. These authors focused their attention precisely on miR-145, which was downregulated in many tumours, thus playing crucial roles in tumour initiation, progression, metastasis, invasion, recurrence, and chemo-radio-resistance. The aim was mainly to study potential miR-145 target genes and to dissect the molecular pathways of tumour pathogenesis associated with those target genes. In this meta-analysis, authors included eight published microarray datasets, in which miR-145 targets were studied in different cell lines when miR-145 was over-expressed. The meta-analysis showed that UNG, FUCA2, DERA, GMFB, TF, and SNX2 were commonly downregulated, while MYL9 and TAGLN were commonly upregulated in prostate, breast, oesophagus, bladder cancer, and head and neck squamous cell carcinoma. These genes are significantly involved in telomere maintenance, DNA binding, and repair mechanisms. Therefore, miR-145 may contribute significantly to tumour pathogenesis in several cancer types and could serve as an important target for cancer therapy. MiRNAs in situ analysis demonstrated that the distribution of miRNA-expressing cells varies in malignant cells, stromal and other cells, in PC tissues. 

#### 1.1.1. Downregulated miR-145 

Recently, Karadag et al. identified how miRNAs allow for differentiating malignant PC from benign prostatic hyperplasia (BPH) [20]. miRNA expressions were determined by qPCR and the miR-145-5p expression level was significantly reduced in PC patients as compared to BPH patients. Additionally, ectopic miR-145-5p expression resulted in significant inhibition of cell proliferation and altered cell morphology, significantly interfering with PC cell migration, revealing an important role of this miRNA in PC as a potential therapeutic target.

**Figure 1:**
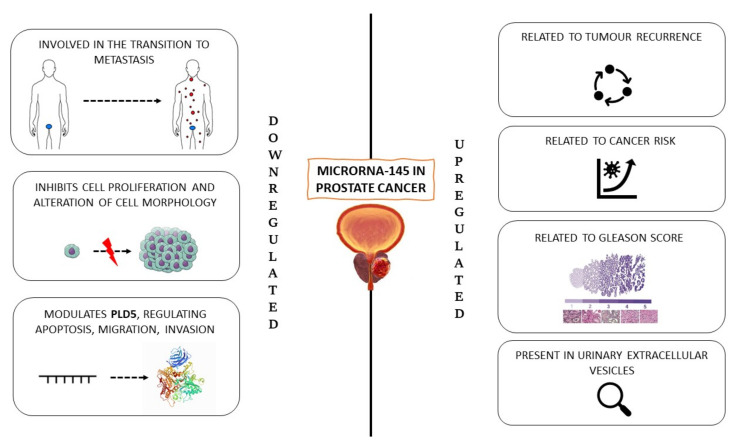
miR-145 in prostate cancer.

Another recent investigation explored the association between miR-145-5p and phospholipase D-5, PLD5, to elucidate their function in regulating PC cell line proliferation [21]. Phospholipase D (PLD) is a transphosphatidylase enzyme [22] whose overexpression is linked to various cellular effects, such as migration, membrane fusion, growth, survival, and metabolism [23]. The dysregulated interaction between PLD and miR-145-5p contributes to numerous pathological conditions, including different kinds of cancers, such as renal, brain, breast, gastric, thyroid, and colorectal cancers [24]. This investigation hypothesised the role of miR-145-5p in suppressing PC by targeting PLD5. The results described significantly reduced levels of miR-145-5p in PC as compared to IPBH tissues. On the other hand, RT-qPCR results indicated high expression of PLD5 in PC cells. Moreover, miR-145-5p was also able to regulate apoptosis, suppressing PC migration, invasion, and metastasis directly modulating PLD5. The assessment of miR-145-5p/PLD5 could represent a novel diagnostic tool to discriminate PC from IPBH patients, representing, at the same time, an alternative therapeutic goal (Figure 1).

Avgeris et al. evaluated the clinical utility of miR-145 for the diagnosis and prognosis of PC [25]. Total RNA was isolated from prostate tissue samples of 73 patients with PC and 64 patients with IBPH. MiR-145 was expressed in a different manner. Downregulation of miR-145 in PC was correlated with a higher Gleason score, advanced clinical stage, larger tumour diameter, and higher prostate-specific antigen (PSA) levels. In the same patients, a higher risk of biochemical recurrence and a significantly shorter disease-free survival (DFS) were found. 

Wach et al. described the diagnostic potential of miRNAs miR-375, miR-143, and miR-145 in prostate cancer from a cohort of 20 cancer samples [26]. Differential expression of miRNAs in situ is cancer-cell associated. MiR-145 and miR-221 were again downregulated. A combination of three miRNAs correctly classified tissue samples with an accuracy of 77.6% with an area under the receiver–operator characteristic curve of 0.810. Their results indicate that combinations of miRNAs are promising biomarkers for the diagnosis of prostate cancer.

#### 1.1.2. Upregulated miR-145

Leite et al. studied the expression of 14 miRNAs in samples from 49 patients treated for PC with radical prostatectomy, finding that the expression of miR-145 is related to tumour recurrence [27]. Karatas et al. investigated the possible involvement of miR-145 in PC progression and found that it was differentially expressed in 41 recurrent and 41 non-recurrent PC tissue samples [28]. Validation showed that miR-145 was reduced in recurrent PC samples but not in a significant manner compared to non-recurrent PC. 

These contradictory findings could be caused by analytical reasons, as different studies calculated the expression of miR-145 by using different normalizers, different kinds of samples, or very short follow-up times that make assessment difficult. 

Additionally, in plasma, miRNA levels may allow for the distinguishing of patients according to disease severity. Shen et al. analysed the plasma of 82 patients with PC [29]. A significant increase in miR-145 expression was observed in patients at an intermediate or high risk as compared to patients with a low-risk score.

Xu et al. investigated the presence of PC-related miRNAs in urinary extracellular vesicles (EVUs) as biomarkers of this neoplasm [30]. Indeed, extracellular vesicles can be detected in body fluids and may serve as biomarkers of the disease. Furthermore, circulating miRNAs in serum and urine may be potential non-invasive biomarkers for PC. Therefore, the expression of four potential miRNA markers of PC, including miR-145-5p in EVUs, was determined in a cohort of 60 patients with PC, 37 patients with benign prostatic hyperplasia (BPH), and 24 healthy controls. MiR-145 was found to be significantly increased in PC patients as compared to BPH patients. MiR-145 was significantly increased in patients with a Gleason Score ≥8 as compared to those with a Gleason Score ≤7, while no significant difference was found between patients with localised and metastatic cancer.

#### 1.1.3. MiR-145/mRNA Network in PC

Presently, patients with PC that are not suitable for radical therapy are often treated with androgen deprivation therapy (ADT) which initially gives a good response. Larne et al. tried to elucidate the mechanism by which deregulation of miR-145 may contribute to PC progression, evaluating an association with androgen receptor (AR) expression [31]. 

They found that this miRNA is essential for the initiation, progression, and transformation of PC into a lethal castration-resistant state. Levels of miR-145 in situ were inversely correlated with the occurrence of metastases, survival, and response to ADT in a well-characterised cohort of PCs. In the same study, the introduction of ectopic miR-145 into PC cells was attempted, generating an inhibitory effect on AR at both transcriptional and protein levels, as well as on its activity and on PSA. The results were verified in clinical prostate samples, demonstrating inverse correlations between miR-145 and AR expression and serum PSA levels. Furthermore, miR-145 was found to be involved in the regulation of androgen-dependent cell growth in vitro. Authors conclude that miR-145 could reduce transformation into the fatal form of PC.

Wang et al. investigated miRNA-mRNA pairings expression in the African American population, which shows higher incidence and mortality rates for PC as compared to the Caucasian population [32]. Authors identified 22 miRNAs specific for African Americans and 18 for Europeans differentially expressed in PC. The analysis performed revealed that the regulation of epidermal growth factor receptor (EGFR) signalling is a critical pathway significantly regulated by African American-specific mRNAs and miRNA-mRNA pairings. One of these regulatory pairs is miR-145/inositol 1,4,5-triphosphate receptor type 2 (ITPR2) (down-up). Recent genome-wide association studies have implicated the ITPR2 gene represents a novel risk locus for renal cell carcinoma [33], while miR-145 is considered a tumour suppressor being downregulated in several tumour types. These findings point out that miR-145 and ITPR2 act as a mutual functional pair promoting invasion and proliferation in African American patients with PC.

A recent study in castration-resistant prostate cancer (CRPC) cell lines showed that miR-145 was downregulated in this subset of the disease [34]. Patients often treated with ADT at its occurrence give a good response; however, almost all of these patients progress from androgen-sensitive prostate cancer (ASPC) to CRPC, which no longer responds to ADT and is characterised by local recurrences and distant metastases. Furthermore, miR-145 targets c-MYC and CDKN1A. C-MYC is an oncogene over-expressed in PC [35] and has been reported to promote CRPC progression [36]. CDKN1A induces cell cycle arrest, which is contrary to the hypothesis that miR-145 induces cell cycle arrest in PC [35]. Another study showed that miR-145 inhibits CDKN1A, but also induces important pro-apoptosis molecules. Therefore, the anti-proliferative effect elicited by miR-145 is mainly due to the activation of apoptosis rather than to cell cycle arrest [37]. This finding supports the hypothesis that a sophisticated miRNA/mRNA interaction network exists in CRPC.

Coarfa et al. described the proteomic fingerprint of a panel of 12 microRNAs that are potently suppressed in metastatic CRPC, including miR-145- 3p, using reverse-phase proteomic arrays (RPPA) [38]. Re-expression of these miRNAs in PC cells suppressed cell proliferation regulating key oncogenic pathways as well as cell cycle, apoptosis, Akt/mTOR signalling, metastasis, and the AR axis. Inverse correlations have been identified between the expression of these miRNAs and early clinical relapse. The results of this study demonstrate that epigenetic silencing of these miRNAs can result in increased AR axis activity and cell proliferation, thus contributing to disease progression. 

Using next-generation sequencing, Goto et al. analysed miRNAs from metastatic CRPC samples [39]. The expression signature of miRNAs was determined by comparing their expression among CRPC, normal prostate tissue, and HSPC. MiR-145-5p and miR-145-3p were upregulated in CRPC; miR-145-3p targeted 4 key genes, namely, MELK, NCAPG, BUB1, and CDK1 in CRPC, predicting survival in patients with PC. This study provided new therapeutic targets for CRPC.

MiR-145 was also detected in endothelial cells of blood vessels and to some extent in stromal tissue (Table 2). In addition, ROC analysis identified miRNA-145 as the more suitable miRNA, able to discriminate between tumours and non-cancerous tissue, correctly classifying them in 71% of cases.

### 1.2. MiR-148

#### 1.2.1. Downregulated MiR-148

**Figure 2:**
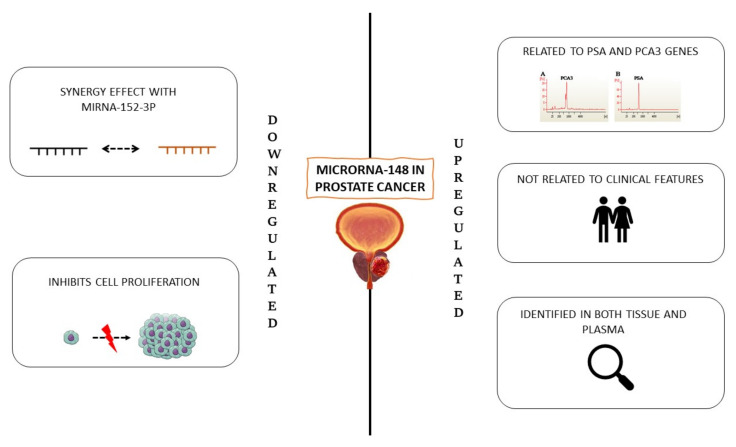
miR-148 in prostate cancer.

Walter et al. investigated the significance of different microRNAs in tumour cells, normal epithelium, and adjacent stroma from biopsies of 37 PC patients. [40]. MiRNAs were extracted for PCR array profiling and used to compare tumour samples with normal epithelium and tumour-adjacent stroma. In high-grade tumours, downregulation of miR-148 was found. Feng et al. evaluated the synergistic effect of miR-148-3p and miR-152-3p in PC [41]. These two miRNAs appeared to be downregulated in PC tumour tissues. Furthermore, miR-148-3p-induced growth inhibition of PC cells was observed both in vivo and in vitro. Therefore, it can be suggested that the miR-148-3p/152-3 p family might act as tumour suppressors in PC (Figure 2).

#### 1.2.2. Upregulated miR-148

In a recent study, the expression levels of three miRNAs and three genes in tissue samples of PC and benign prostatic diseases (benign prostatic hyperplasia, prostatitis) were evaluated as candidates to detect this neoplasm [42]. Results showed a positive correlation between miR-148b-3p and PSA and PCA3 gene expression, the latter being well-established biomarkers in PC. The expression of miR-148b-3p was not correlated to clinical features, such as age and weight, as observed for the other miRNAs analysed, suggesting that it is a potential biomarker for detecting this disease.

In another study, expression profiles of PC miRNAs were determined by deep sequencing [1]. Some miRNAs were clearly differentially expressed according to the tumour stage as compared to the corresponding healthy, non-malignant prostate tissue. Quantitative real-time PCR (qRT-PCR) analysis of 40 PC samples revealed significant upregulation of miR-148 and miR-145 as compared to normal tissues. 

In a study published in 2019, miRNA profiling was performed in plasma and tissues of PC patients, in the attempt to identify a single candidate miRNA as a biomarker [43]. The results revealed that miR-148a-3p was upregulated in tissue and plasma samples, respectively (Table 3).

### 1.3. MiR-185

#### 1.3.1. Downregulated MiR-185

Hellwinkel et al. [44] analysed histologically normal prostate tissue samples from 31 patients with PC and two cancer-negative control groups, 14 and 17 individuals with unpredicted or elevated PSA levels, respectively. In the pilot study, they quantified the levels of eight selected miRNAs in normal prostate tissue to assess their potential role as markers of tumours foci elsewhere in the prostate.

Seven miRNAs (miR-124a, miR-146a & b, miR-185, miR-16, and let-7a & b) showed significant differential expression in normal prostate tissue of men with PC as compared to both cancer-negative control groups. Within this group, only four miRNAs, including miR-185, were confirmed to be significant discriminators between PC patients and the cancer-negative group with elevated PSA levels. 

It is still unclear how miR-185 can regulate tumour processes in general or in PC, and this study clearly confirms the role of this miRNA as a tumour suppressor [45,46,47]. 

**Figure 3:**
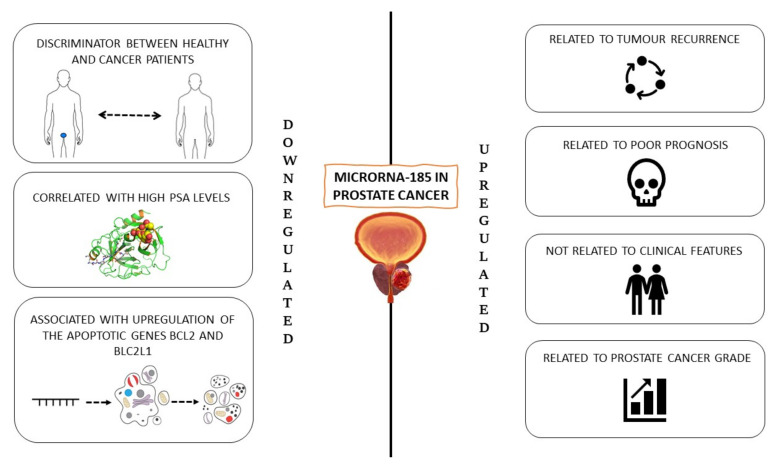
miR-185 in prostate cancer.

Ostadrahimi et al. demonstrated that, among the 12 candidate miRNAs, miR-185 expression was markedly downregulated in PC tissues and cell lines [48]. Furthermore, the downregulation of this miRNA was associated with the upregulation of the anti-apoptotic genes BCL2 and BCL2L1. Therefore, an inverse association between this miRNA and two anti-apoptotic genes may be considered for interventional miRNA therapy in PC (Figure 3).

#### 1.3.2. Upregulated miR-185

Kristensen et al. demonstrated that a miRNA signature with significant independent prognostic value was detectable in three cohorts of PC patients. Novel diagnostic and prognostic classifiers for prostate cancer were identified by genome-wide microRNA profiling [49]. The analysis identified several novel deregulated miRNAs among non-malignant tissue (NM), PC samples, and aggressive PC subgroups. They also developed and validated a novel 13-miRNA diagnostic classifier with high sensitivity and specificity and a novel 3-miRNA prognostic classifier (miR-185-5p + miR-221-3p + miR-326) in a cohort of 126 patients, predicting time to biochemical recurrence, independently from routine clinicopathological variables after radical prostatectomy.

Another experimental design, involving 752 miRNAs in 13 NM and 134 PC tissue samples (cohort 1), selected 93 diagnostic/prognostic miRNAs for validation in 2 independent patient sets (cohort 2 with 19 NM and 138 PC; cohort 3 with 28 NM and 113 PC samples). In total, 8 miRNAs, including miR-185-5p, were associated with clinicopathological measures of PC aggressiveness in multiple cohorts; however, they were mostly associated with biochemical recurrence. In cohort 2, upregulation of miR-185-5p in PC was confirmed. High expression of miR-185-5p has been associated with poor prognosis in radical prostatectomy cohorts, although previous studies have shown that miR-185 inhibits proliferation, migration, and invasion and induces apoptosis in some PC cell lines [50,51,52]. This inconsistency can be explained by the many differences specific to each cell type. Indeed, many miRNAs behave as oncogenes in some cells and as tumours suppressors in others [53]. Furthermore, previous studies [50,51,52] do not distinguish between miR-185-5p and miR-185-3p. MicroRNAs are linked to factors associated with aggressive PC, such as biochemical recurrence, and metastasis. McDonald et al. investigated whether circulating miRNAs in plasma could be used as diagnostic biomarkers for PC [54]. Where 66 patients with newly diagnosed PC were classified into two risk groups: 40 patients with low-grade (Gleason score (GS) 6 or 7 and serum PSA < 20 ng/mL) and 26 patients with high-grade PC (GS ≥ 7 and serum PSA ≥ 20 ng/mL). In high-grade PC cases, the expression of miR-185 was higher than in low-grade cases, indicating them as promising biomarkers of PC. The result changed completely after multiple comparisons and after adjustment for age, thus there was no circulating miRNA associated with high-grade PC. In a very recent study, Gurbuz et al. evaluated the expression of 30 miRNAs in PC in a clinic-pathological perspective to identify potential biomarkers in the diagnosis and progression of PC at different stages [55]. Whole blood samples were divided into 25 controls, 25 PCs, and 40 patients with metastatic PC and analysed using the quantitative real-time PCR. The study demonstrated an association between the expression levels of the 30 miRNAs included in the study and PSA levels. In patients with PSA ≥ 10, miR-185-5p expression levels were statistically significant increases (*p* < 0.05), as well as in patients with PSA < 10, in those with metastatic PC and PSA > 20, and for patients with PSA ≤ 20 and PSA> 20. The stepwise increases in many miRNAs, including miR-185 -5p, being statistically significant in patients with local, locally advanced, and metastatic PC (*p* < 0.05), suggesting that this miRNA may be used as biomarkers in disease prognosis (Table 4). 

### 1.4. Involvement of Stem Cells in the Development of Prostate Cancer

Increasing evidence suggests that cancer stem cells (CSCs) in PC tissue are responsible for treatment failure due to their key role in metastasis, epithelial-mesenchymal transition, and drug resistance [56,57]. Moreover, aberrant miRNA expression has been reported to play an important role in maintaining PCSC associated with drug resistance, metastasis, and therapeutic failures [58]. Therefore, we described the recent reports on PCSC playing critical roles in PC origin and metastasis and the regulatory role of miRNAs in PCSCs. 

Stem cells are critical in maintaining the homeostasis of the entire organism, with an extensive ability to self-renew and differentiate into tissue-specific phenotypes. Due to their pluripotency and self-renewal properties, stem cells have gained great importance in therapy [59]. Recent studies suggest the role of stem cells in cell turnover and morphogenesis in the normal prostate. The prostate is a highly complex gland of the male urogenital system, composed of multiple differentiated cell types, including basal, luminal, and neuroendocrine cells, along with undifferentiated stem cells [60]. In addition to prostate basal epithelial stem cells (PBSCs), also stromal stem cells (SSCs) within the stromal cell layer have also been found in the prostate. Like other tissues, PSCs are localised in a defined microenvironment, which is known as their “niche.” The niche influences stem cell fate, either by keeping them in a quiescent state or inducing proliferation and differentiation [61]. These subpopulations can be characterised for specific surface markers, such as CD34, vimentin, CD44, CD117, and CD90, and exhibit the ability to differentiate into several phenotypes. Dysregulation of stem cell behaviour and their capability to control self-renewal can lead to tumorigenesis in the human prostate, by hyperproliferation of epithelial and stromal cells in the transition zone of the prostate gland [62,63,64]. Cell proliferation is the most important hallmark of cancer, and its abnormality is the main cause of tumorigenesis. Cell cycle progression is controlled by intracellular programs and extracellular signal molecules to achieve the balance between the promotion/suppression of cell proliferation. When cell proliferation and growth are out of control, cells may become cancerous. The tumour cell population with self-renewal capabilities includes the so-called CSCs, which in the case of the prostate are referred to as PCSCs. In pathological conditions there is a lack in the regulation of signalling pathways that maintain proper stem cell physiology, leading to hyperplasia of the prostate stroma [65,66]. The tumour microenvironment and the crosstalk between the epithelial niche and mesenchymal cells seem to be involved in the development of PC. The exhausted medium by human HepG2 and MCF7 cell lines can modulate stem cell fate by direct activating specific epigenetic modifications, increasing the expression of stemness-related genes, cell proliferation, and autophagy [67,68]. Genetic manipulation of stromal cells can lead to tumour formation, as well as the possible reprogramming of adult differentiated cells to an embryonic-like state, indicating that the microenvironment is a key player in the control of oncogenic cell signals and in the generation of cancerous conditions [69]. Chromosomal abnormalities, changes in transcriptional control, epigenetic modifications, and defects in the mechanism of miRNAs biogenesis represent some of the mechanisms which can be involved in the pathogenesis of PC [70]. Within this context, miRNAs play a key role in stem cells in the reprogramming process, maintaining stem cell pluripotency and differentiation and modulating the expression of some key genes, as CD44, Oct4, Sox2, c-Myc and Klf4 [58,71,72]. It is already demonstrated that miRNAs regulate PCSC stemness both through a direct regulation of transcription factors and stemness-related markers and indirectly, by influencing the epithelial-mesenchymal transition (EMT) [72]. 

Overexpression of miR-133a/b and miR-145 has been shown to inhibit cell proliferation and induce cell apoptosis in several cancer types, with cell cycle arrest in the G2/M phase, through regulation of the pro-apoptotic gene TNFSF10. In addition, miR-145 directly regulates the pluripotency factors OCT4, SOX2, and Klf4, maintaining stem cell pluripotency [73]. Other authors confirmed the role of miRNAs in controlling several oncogenic signalling pathways, such as Notch, wingless (WNT)/β-Catenin, and nuclear factor kappa-light-chain-enhancer of activated B (NF-kB) [58]. Furthermore, miR-145 is significantly downregulated in PC and plays an important role in the regulation of PC bone metastasis, by a mechanism that is still not fully understood. Hence, miR-145 might act by inhibiting the progression of bone metastases by increasing the total number of cells that undergo apoptosis and mediating cell epithelial-mesenchymal transition (EMT) [74]. Additionally, miR-143 and miR-145 also inhibit cell viability and colony formation and tumorspheres of PC-3 cells from PC bone metastases by repressing the expression of CSC markers and stemness factors including CD133, CD44, Oct4, c-Myc, and Klf4 [74]. MiR-148b is a tumour suppressor that can regulate oncogenes related to invasion, apoptosis, and proliferation. Several classes of DNA methyltransferases (DNMTs) and histone deacetylases (HDACs) are among the main targets of mir-148 in PC. It has also been found that an increase in miR-148a-3p in serum may be a potential biomarker in PC [75]. MiR-148a expression is also altered during the progression of the various stages of cancer, inhibiting metastasis and acting synergistically with miR-152-3p suppressing Klf4, and the stemness markers SOX2, OCT4, and Nanog. Overexpression of miR-148a is also able to significantly counteract migration and invasion, increasing apoptosis of tumour cells [76,77]. Furthermore, miR-185 inhibits tumorigenicity, cell growth, migration, and invasion by inducing caspase/PARP-mediated apoptosis. In addition, overexpression of miR-185 blocks PC cell proliferation and angiogenesis of HUVECs by suppressing the ALK4-signalling cascade [52,78]. Overexpression of miR-185 suppresses osteosarcoma (OS) cell proliferation and migration through E2F1 and DNMT1 pathways, and by modulating HK2 protein expression in OS cells. In addition, miR 185 suppressed OS cell glucose metabolism and inhibited the Wnt/β-catenin pathway in colorectal cancer [79]. The identification of other direct and indirect mechanisms of action of miRNAs and their ability to specifically modulate PCSC stemness could therefore represent a new molecular therapeutic target for prognosis and targeted therapy for human PC bone metastases.

## 2. Materials and Methods

An electronic search was performed on Medline databases (PubMed interface) and for articles published from 2011 to 14 September 2021 (10-year interval). The terms used for the search were “miR-145 AND prostate cancer”, or “miR-148 AND prostate cancer”, or “miR-185 AND prostate cancer”, or “stem cells AND prostate cancer”, or “miRNA AND stem cells”. Inclusion criteria were (a) full-text articles reporting studies on a miRNA of interest in adult patients with prostate cancer; (b) English; (c) meta-analysis with data on that topic; (d) studies approved by an ethics committee and performed in accordance with the principles of the Declaration of Helsinki. The electronic search yielded 131 results; after exclusion of duplicates, irrelevant articles, reviews, papers not in English, or missing data, 73 articles were included for review. The total number of patients in the retrieved studies was 1545. Abstracts were selected and selected full-text articles were reviewed. This article is based on previously performed studies and contains no new studies with human or animal participants performed by any of the authors.

## 3. Conclusions

PC is the second most common cause of cancer death in men. The differential diagnosis of PC is currently based on prostate biopsy, after the digital rectal examination (DRE), plasma PSA levels, and further supported by transrectal ultrasound. However, better diagnostic tools are desperately needed. More recently, the advent of high-throughput molecular technologies has enabled the discovery of new biomarkers. Kallikrein-related peptidase 2 (KLK2) is one of the biomarkers that are highly expressed in prostate cancer but not in normal prostate tissue. Furthermore, the long non-coding intronic RNA, prostate cancer gene 3 (PCA3), has been identified as a highly specific prostate cancer biomarker that is widely expressed in prostate cancer tissues as compared to benign prostate disorders. However, the diagnostic utility of these biomarkers is relatively poor and needs to be supported by other novel molecular biomarkers. Furthermore, due to delayed diagnosis, treatment success decreases, and the frequency of recurrence increases. Therefore, the identification of new biomarkers for the efficient diagnosis and treatment of prostate cancer is necessary. Therefore, miRNa-145, 148, and 185 play crucial roles in the pathogenesis and progression of prostate cancer. PCSCs are involved in many processes during prostate cancer development. Therefore, the mutual relationships between these two players seem to be interesting and important. The miRNAs examined play a key role in prostate cancer pathogenesis and in the regulation of PCSC self-renewal, proliferation, and differentiation. The effect of miRNAs on the behaviour of PCSCs during tumour progression could be further exploited to unravel novel diagnostic tools able to immediately identify the different stages of the pathological condition within the different groups of patients.

## Figures and Tables

**Table 1:** Frequently reported dysregulated miRNAs in prostate cancer.

MiRNA	Expression	Sample Types, Author, Year		
		Tissue	Serum/Plasma	Urine
miR-21		Szczyrba, 2018; Wach, 2012	NA	NA
	Up	Zedan, 2018; Kumar, 2018	Porzycki, 2018; Kotb, 2014; Egidi, 2013; Zedan, 2018; Agaoglu, 2011; Mello Grand, 2018; Gao, 2016; Endzelinš, 2017—EVs	Ghorbanmehr, 2019; Foj, 2017
miR-125b	Down	Zedan, 2018; Schaefer, 2010	NA	Fredsoe, 2018
	Up	Walter, 2013; Song, 2015	Mitchell, 2008; Zedan, 2018	NA
miR-141	Down	NA	Kachakova, 2014	Fredsoe, 2018
	Up	Szczyrba, 2010; Kumar, 2018; Nguyen, 2013; Brase, 2014; Kelly, 2015	Mitchell, 2008; Porzycki, 2018; Cheng, 2013; Guo, 2018; Hao, 2016—EVs Bryant, 2012—EVs	Ghorbanmehr, 2019; Foj, 2017
miR-143	Down	Szczyrba, 2010; Wach, 2012; Zedan, 2018; Kumar, 2018; Martens Uzunova, 2012	NA	Stuopelyte, 2016 Rodriguez, 2017—EV
	Up	NA	Nitchel, 2008; Zedan, 2018	NA
miR-145	Down	Szczyrba, 2010; Wach, 2012; Porkka, 2007; Ozen, 2008; Kang, 2012; Zedan, 2018; Kurul, 2019; Schaefer, 2010; Larne, 2015, Kelly, 2015; Martens-Uzunova, 2012; Yfantis, 2008; Avgeris, 2013; Larne, 2013; Karatas, 2014 Wang, 2015, Zhu, 2015 Goto, 2017, Coarfa, 2016 Karadag, 2021, Liu, 2021 Leite, 2011	NA	NA
	Up	NA	Shen, 2012	Xu, 2017
miR-148	Down	Feng, 2019, Walter, 2013 Arámbula-Meraz, 2020	NA	Stuopelyte, 2016
	Up	Szczyrba, 2010; Martens-Uzunova, 2012; Stuopelyte, 2016; Lichner, 2015; Hart, 2013	Dybos, 2018; Al-Qatati, 2017; Paunescu, 2019	NA
miR-182	Down	NA	NA	NA
	Up	Wach, 2012; Schaefer, 2010; Yfantis, 2008; Tsuchiyama, 2013; Costa-Pinheiro, 2015; Casanova-Salas, 2014	NA	NA
miR185	Down	Hellwinkel, 2013, Ostadrahimi, 2018	Mc Donald, 2019, Gurbuz, 2020	NA
	Up	Kristensen, 2016	NA	NA
miR-200c	Down	Szczyrba, 2010; Wach, 2012; Yfantis, 2008	NA	Fredsoe, 2018
	Up	NA	Cheng, 2013 De Souza, 2017 Endzelinš, 2017—EVs	NA
miR-205	Down	Scahefer, 2010; Martens-Uzunova, 2012; Yfantis, 2008; Tsuchiyama, 2013; Verdoodt, 2013; Kalogirou, 2013	Guo, 2018 Osipov, 2016	Fredsoe, 2018
	Up	Walter, 2013	NA	NA
miR-221	Down	Szczyrba, 2010; Wach, 2012; Zedan, 2018; Kurul, 2019; Schaefer, 2010; Yfantis, 2008; Porkka, 2007; Tsuchiyama, 2013; Casanova-Salas, 2014; Kneitzm, 2014	NA	Fredsoe, 2018
	Up	Song, 2015	Kotb, 2014 Agaoglu, 2011	NA
miR-222	Down	Wach, 2012; Schaefer, 2010; Martens-Uzunova, 2012; Porkka 2007; Tsuchiyama, 2013	NA	Fredsoe, 2018
	Up	NA	NA	NA
miR-375	Down	Szczyrba, 2010; Wach, 2012; Schaefer, 2010; Nguyen, 2013; Brase, 2011; Stuopelyte, 2016; Yfantis, 2008; Costa-Pinheiro, 2015; Haldrup, 2014; Nam, 2018	Kachakova, 2014	NA
	Up	NA	Porzycki, 2018; Nguyen, 2013; Brase, 2011; Cheng, 2013; Haldrup, 2014; Wach, 2015; Zedan, 2018; Gao, 2016; Endzelinš, 2017; McDonald, 2018; Huang, 2015—EVs Bryant, 2012—EVs	Foj, 2017; Stuopelyte, 2016
let-7a	Down	Szczyrba, 2010; Wach, 2012; Kelly, 2015; Porkka, 2007; Tian, 2015	Endzelinš, 2017—EV	Fredsoe, 2018
	Up	Haldrup, 2014	Mello-Grand, 2018	NA

Note: Data were obtained from several research studies based on a meta-analysis by Song et al. [12] and Bertoli et al. [13]; EV: Extracellular vesicle.

**Table 2:** Main characteristics of the articles selected and revised for miR-145.

Author, Year, Place	Expression	Prostate Cancer Patients (n)	Negative Control (n)	Samples Type	Reference
Leite, 2011, Brazil	Downregulation	49	10	Tissue	[27]
Shen, 2012, USA	Upregulation	82		Plasma	[29]
Wach, 2012, Germany	Downregulation	76		Tissue	[26]
Avgeris, 2013, Greece	Downregulation	73	64	Tissue	[25]
Leite, 2013, Brazil	Downregulation	63		Tissue	[17]
Hart, 2013, Germany Karatas, 2014, Turkey, USA	Downregulation	40	40	Tissue	[1]
	Downregulation	82		Tissue	[28]
Larne, 2015, Sweden	Downregulation	49	25	Tissue	[31]
Wang, 2015, USA	Downregulation	35		Tissue	[32]
Zhu, 2015, China	Downregulation	5		Cell lines	[34]
Coarfa, 2016, USA	Downregulation	113	28	Tissue	[38]
Goto, 2017, Japan	Downregulation	34	19	Tissue	[39]
Xu, 2017, China	Upregulation	60	24	Urine	[30]
Karadag, 2021, Turkey	Downregulation	18	18	Cell culture	[20]
Liu, 2021, China	Downregulation	18	18	Cell culture	[21]

**Table 3:** Main characteristics of the articles selected and revised for miR-148.

Author, Year, Place	Expression	Prostate Cancer Patients (n)	Negative Control (n)	Samples Type	Reference
Walter, 2013, USA	Downregulation	40	20	Tissue	[40]
Hart, 2014, Germany	Upregulation	40	40	Tissue	[1]
Feng, 2019, China	Downregulation	42	42	Tissue	[41]
Paunescu, 2019, Romania	Upregulation	14	15	Plasma	[43]
Arámbula-Meraz, 2020, Mexico	Downregulation	12	12	Tissue	[42]

**Table 4:** Main characteristics of the articles selected and revised for miR-185.

Author, Year, Place	Expression	Prostate Cancer Patients (n)	Negative Control (n)	Samples Type	Reference
Hellwinkel, 2013, Germany	Downregulation	31	31	Tissue	[44]
Kristensen, 2016, Denmark	Upregulation	385	60	Tissue	[49]
Ostadrahimi, 2018, Iran	Downregulation	30	30	Tissue and cell lines	[48]
Mc Donald, 2019, USA	Upregulation	66	/	Plasma	[54]
Gurbuz, 2020, Turkey	Upregulation	75	25	Blood	[55]

## Data Availability

Not applicable.
